# Partial Resection of the Lung for Abscess

**Published:** 1918-10

**Authors:** 


**Partial Resection of the Lung Because of Abcess.** Raymond Grégoire, *Bulletin de la Société de Chirurgie,* August, 6, 1918.

Undoubtedly the great number of chest wounds treated during the war have given rise to a bolder technic in the surgery of the lung.

Observations on partial resection are rare enough, the author believes, to warrant the publication of notes on the following case:

A colonial soldier from Algeria entered the hospital of the 18th region on the 14th of June 1918, with a diagnosis of slight dyspnea. The electro-vibrator and radiography revealed a German bullet on the right axillary line toward the seventh intercostal space. All the lower half of the hemothorax was darkly discolored owing to thoracic extravasion. The eighth rib was resected in a wide excision, and a fairly important sero-hematic effusion of the pleura was found and removed. At the lower extremity of the lobe, there was a large tumefaction. The tumor was dark red with yellowish patches, the lung being only a little darker that usual. A puncture showed the tumor to be an abcess grown around the projectile. The whole abcess zone was resected in the parenchyma; the blood was black, and the abcess yielded a quantity of pus. The point of the bullet could then be seen to have perforated the wall of the cavity. There was, however, moderate bleeding.

Courcoux had already found, when sectioning the edge of the lung in a dog, that it bled very little. Suture was made afterwards without fear of hemorrhage. The lung was replaced in its cavity, and the wall also sutured.

On the 19th of June the patient was slightly oppressed. A pronounced pneumothorax was observed with a small quantity of liquid in the pleura. A puncture was made evacuating the air and the liquid. A month afterward the patient was completely cured.

This observation proves the necessity of extracting projectiles lodged in the lung.

Undeniably, immediate intervention presents great dangers, but when the traumatic lesions are healed the extirpation of the projectile is imperative. Besides functional troubles, projectiles may also cause ulceration or even very much delayed abcesses. Before intrapulmonary abcesses the most simple technic is the suture of the lung to the parietal pleural, then the opening of the collection and its draining from without. Gregoire, however, decided to resect at the same time the abcess and the pulmonary parenchym, which perhaps may be contamined also. He does not recommend this as a regular form of treatment for pulmonary abcesses, but his observation is of particular interest because the resection involved a rather large portion of the lung. As it happened, the abcess occupied a part of the lung where resection was possible, but such is not always the case with the anatomy of the organ.

Danger of hemorrhage is very much lessened since after pneumothorax, or effusion, there is a considerable diminution of the circulation. The most difficult attainment is to avoid the infiltration of air in the pleural cavity. Grégoire, indeed, thinks that pneumothorax under tension owes its origin to smaller bronchi having been sectioned and left gaping. Air, in the present case, had filtered into the pleural cavity either during coughing or breathing, and the pressure increased progressively since there was no outlet for the air to escape.

It would be interesting to know how the space left by the resected lung is filled. So far, radioscopy has not permitted to say ; perhaps the diaphragm rises slightly. At any rate, the fact remains that the patient did not feel any discomfort.

